# Effects of Stocking Density in the Pen and Lairage Time on Blood Stress Indicators, Skin Lesion Scores, and Pork Meat Quality

**DOI:** 10.3390/ani15050634

**Published:** 2025-02-21

**Authors:** Luana Torres da Rocha, Paulo Levi de Oliveira Carvalho, Janaína Paolucci Sales de Lima, Liliana Bury de Azevedo, Silvana Texeira Carvalho, Jansller Luiz Genova, Luigi Faucitano

**Affiliations:** 1 Department of Animal Science, Universidade Estadual do Oeste do Paraná, Marechal Cândido Rondon 85960-000, PR, Brazil; luana.torres.13@gmail.com (L.T.d.R.); paulolevi@yahoo.com.br (P.L.d.O.C.); liliana.bury@hotmail.com (L.B.d.A.); silteixeira@gmail.com (S.T.C.); 2 Department of Animal and Plant Production, Universidade Federal do Amazonas, Manaus 69080-900, AM, Brazil; paolucci@ufam.edu.br; 3 Department of Animal Science, Universidade Federal de Viçosa, Viçosa 36570-900, MG, Brazil; jansller.genova@ufv.br; 4 Agriculture and Agri-Food Canada, Sherbrooke Research and Development Centre, Sherbrooke, QC J1M 0C8, Canada

**Keywords:** pigs, lairage time, stocking density, stress, skin lesions, meat quality

## Abstract

The objective of this study was to assess the effects of stocking density in the pen and lairage time before slaughter on blood stress indicators, skin lesions, and pork meat quality traits. A total of 1920 immunocastrated male pigs were shipped to a slaughterhouse. In order to maintain the proposed stocking densities, 72 pigs out of the 1920 animals were removed across loads from the experiment. The remaining 1848 pigs were randomly assigned to two different stocking densities in the lairage pen (0.42 m^2^/100 kg and 0.66 m^2^/100 kg) and two lairage times (2 h and 6 h). A total of 360 pigs were selected for the analysis of exsanguination blood glucose, creatine kinase, lactate dehydrogenase, cortisol concentration, hematocrit percentage, and carcass skin lesion scores, whereas meat quality was assessed in 240 loin muscles. Except for blood hematocrit percentage, the interaction stocking density × lairage pen had no effect on any parameter (*p* > 0.10). When compared to a lower stocking density (0.66 m^2^/100 kg), pigs kept at a higher stocking density (0.42 m^2^/100 kg) tended to present lower blood LDH concentrations (*p* = 0.06) and higher blood cortisol concentrations (*p* = 0.08) at slaughter and had greater carcass skin lesion scores (*p* < 0.001) and drier (lower drip loss) loin muscles (*p* = 0.04). Increased blood hematocrit percentage was found in pigs kept in lairage at a higher stocking density for 6 h when compared with the other lairage conditions (*p* < 0.01). Overall, stocking density, in particular when it was high, influenced more pigs’ stress conditions at slaughter and carcass and meat quality than the time spent in lairage.

## Simple Summary

This study aimed to assess the impact of stocking density in the lairage pen and rest duration before slaughter on blood stress indicators, skin lesion scores, and the quality of pork meat. A total of 1848 immunocastrated male pigs were allocated into two groups based on two stocking densities (0.42 m^2^/100 kg and 0.66 m^2^/100 kg) and two lairage time intervals (2 h and 6 h). We analyzed various blood parameters and evaluated carcass skin lesions, with meat quality being assessed in 240 loins. The results showed that a higher stocking density was associated with reduced exsanguination blood lactate dehydrogenase (LDH) concentrations and greater cortisol concentrations. This latter result may be associated with the increased skin lesion scores and drier loins. Pigs housed at a higher density for 6 h showed increased blood hematocrit percentages. Overall, the findings suggest that stocking density, especially at higher levels, has a more pronounced effect on stress indicators and meat quality than lairage duration.

## 1. Introduction

The optimization of lairage or rest conditions at the slaughterhouse (i.e., pen layout, rest time, housing, and environment) prior to slaughter has the objectives of letting pigs recover from transport, handling stress, and producing pork carcasses and meat of optimal and consistent quality [1]. When these conditions are not controlled, pigs are prevented from resting properly, resulting in an increased proportion of dead-in-pen pigs [2,3], greater fatigue (as assessed by blood lactate and creatine kinase concentrations, heart and respiration rate, and body temperature [4,5]), a higher incidence of pale, soft, and exudative (PSE) or dark, firm, and dry (DFD) pork meat [6,7,8], and higher skin lesion scores and carcass condemnations [9,10].

These effects may be exacerbated by the practice of mixing groups of unfamiliar pigs in the lairage pen, resulting in increased levels of mounting and aggressive behaviors and higher skin lesion scores [11,12], in addition to a long-term (up to 8 h after mixing) rise in body temperature and plasma cortisol concentration [13,14] and a higher proportion of DFD pork meat due to muscle glycogen exhaustion [12,15].

Research has shown that the fighting rate in mixed groups of pigs during lairage can be controlled by reducing the size of the group (from 30 to 15 pigs) [16] or increasing/ decreasing the space allowance in the pen (from approx. 3 to 1 pig/m^2^ or from 0.85 to 0.42 m^2^/pig) [17,18]. However, between the two strategies, it appears that stocking density has a greater positive impact on pigs’ aggression than group size [17,18]. Recently, Song et al. [19] showed a higher proportion of standing and fighting and more exudative loins in pigs kept at a lower stocking density (<0.50 vs. >0.83 m^2^/pig) and at a higher lairage ambient temperature (>24 °C vs. <10 °C). Furthermore, it has been suggested that the impact of space allowance in the pen on fighting behavior can be modulated according to rest time [20], as most fighting is usually observed during the first 0.5–1 h of lairage [17,18]. Weeks [20] proposed a stocking density of 0.42 m^2^/pig for short lairage (<3 h), with the objective of limiting the social interactions to the few pigs nearby rather than spreading them across the entire pen, while more floor space (0.66 m^2^/pig) should be allowed for rest times of more than 3 h to let pigs lie down and move around. Nevertheless, these recommendations are not science-based, and the risk of increasing fighting by keeping pigs in the pen with a larger space allowance and for a longer period cannot be ruled out. Due to the additive effect of fasting, there is evidence that fighting rates increase over time (up to 22 h) during rest prior to slaughter [21], resulting in an increased risk of blemished carcasses [10,17]. The combined effects of feed deprivation and fighting can also result in muscle glycogen exhaustion and a higher proportion of DFD or DFD-like meat [7,21,22].

The objective of this study was to validate the efficiency of the recommended space allowances (0.42 and 0.66 m^2^/pig) according to two rest times (2 h and 6 h) through the evaluation of their impacts on the physiological state of pigs at slaughter (based on blood stress indicators), carcass skin lesion scores, and pork meat quality.

## 2. Materials and Methods

All experimental procedures performed in this study were approved by the institutional committee on the use of production animals (Protocol # P013/2023) of the Universidade Estadual do Oeste de Paraná (UNIOESTE; Marechal Cândido Rondon, PR, Brazil).

### 2.1. Animals and Treatments

A total of 1920 immunologically castrated (VIVAX^®^, Zoetis, São Paulo, SP, Brazil) male pigs (144 ± 11 kg) of the same genetics (progenies of Landrace × Large White sows sired with Pietrain boars) were shipped from five commercial finishing farms, all of which had similar design, housing, feeding, handling systems, and distance from the slaughterhouse but different sizes (Table 1).

Pigs were transported to a commercial slaughterhouse (Frimesa Cooperativa Central, Medianeira, PR, Brazil) through 24 shipments over six weeks (4 shipments/farm/week). Because of its larger size (4500 pigs), one farm (F5) made two shipments over two separate weeks (4 shipments/week, 8 shipments in total), while only 4 loads were shipped from each of the remaining 4 farms during each of the remaining 4 weeks (one farm/week). The description of each farm size and distribution of loads by week are shown in Table 1.

Shipments (approx. 60 min or 50 km) were carried out in the summer in early morning (between 03.00 a.m. and 06.00 a.m.) using four similar double-deck, passively ventilated trailers (Randon Triel–HT, Erechim, SC, Brazil), transporting five pigs in 16 compartments (8 compartments/deck; total of 80 pigs per load) at a density of 235 kg/m^2^. Each shipment/truck load represented a replicate of the lairage treatment. The trailers were driven by different certified drivers.

Feed was withdrawn from all pigs for 10 h before transport and from 13 h to 19 h before slaughter, depending on the applied lairage time. At each farm, pigs were loaded onto the vehicle in groups of 5 to 6 pigs by a trained handling team using plastic bottle rattles.

On arrival at the slaughterhouse, the different loads were unloaded at approximately 20 min intervals using hands and plastic boards. In order to maintain the proposed stocking densities, 72 pigs out of the 1920 animals were removed across loads from the experiment. The remaining 1848 pigs were randomly selected and assigned to one of four groups (77 pigs/group, totaling 308 pigs/week) according to a randomized 2 × 2 experimental design, based on the following lairage treatment combinations (one group per combination): stocking density of 0.42 m^2^/100 kg or 0.66 m^2^/100 kg and 2 h of rest and stocking density of 0.42 m^2^/100 kg or 0.66 m^2^/100 kg and 6 h of rest. Stocking density was applied using pens of different dimensions, i.e., two of 74.2 m^2^ and two of 47.2 m^2^ for the lower and higher stocking density treatments, respectively. The 2 h lairage corresponded to the recommended time needed to complete the pigs’ physiological recovery (according to the return of cortisol to the rest concentrations in the blood at slaughter) from the stress of handling and transport before slaughter [23]. The 6 h lairage time reproduced the longest time used at the collaborating slaughterhouse.

The lairage area featured ventilation fans and water misters for the control of ambient temperature (24.8 ± 6 °C) and pigs’ thermal comfort during rest, respectively. In each pen, pigs had access to water supplemented with an electrolyte solution consisting of sodium (77.2 g/kg), chlorine (61.0 g/kg), potassium (34.9 g/kg), vitamin C (6600 mg/kg), and dextrose (25.0 g/kg) (Alto Desafio^®^, Sanex, Curitiba, PR, Brazil), provided through 12 nipple drinkers per pen. Feed was not provided, as rest time in lairage was shorter than 12 h [24]. Each pen was also enriched with two plastic toys (MS Schippers, Ribeirão Preto, SP, Brazil) hanging from a metal chain attached to the ceiling.

At the end of lairage, pigs were driven out of their pen using boards and plastic bottle rattles and then moved to a CO_2_ gas stunner (Butina^®^ Backloader G3 RelaX, Marel, Holbæk, Denmark) using an automatic push gate system at different times (from 05:30 a.m. to 12:30 p.m.) according to the treatment. Pigs were stunned in sub-groups of 5 and, at the exit of the stunner, were shackled, vertically hoisted onto a rail, and bled-out within 30 s.

After slaughter, the carcasses were processed through dehairing, singing, evisceration, and splitting into two halves, and they were ultra-chilled (−18 °C to −8 °C for 1.5 h) and chilled (2 °C to 5 °C for 22 h) following the slaughter plant’s procedures.

### 2.2. Blood Sampling and Analysis

A total of 360 pigs (15 pigs/replicate/treatment) were randomly selected during the exsanguination stage for blood collection in polypropylene flasks (100 mL) for the analysis of plasma glucose, serum creatine kinase (CK), serum lactate dehydrogenase (LDH), serum cortisol, and hematocrit.

The collected blood was divided into three glass tubes (Labingá, Maringá, PR, Brazil) with different additives for the specific biochemical analyses. The first tube containing EDTA K3 anticoagulant (model TV040EK, Vacuplast Collect Line, Ma’anshan, China) was used for the analysis of the blood hematocrit percentage, the second one containing sodium fluoride (model 50204, Labor Import, Jaipur, India) was used for the analysis of the blood glucose concentration, and the third tube containing clot activator (model TV040SGC, Vacuplast Collect Line, Ma’anshan, China) was used for the analysis of blood LDH, CK, and cortisol concentrations. Similar blood sample collection and handling methods were applied prior to analysis in previous studies [25,26,27]. All tubes were then placed in a Styrofoam box filled with ice (4 °C) and were shipped to the SBS Agronomic and Veterinary Analysis Laboratory (Cascavel, PR, Brazil) for the biochemical analyses according to standard procedures normally applied during preslaughter trials at commercial abattoirs [25,28].

Immediately after delivery at the laboratory, the blood hematocrit percentage was analyzed through the microhematocrit method (BC-2800Vet, Mindray, Shenzhen, China), where blood samples were placed in capillary tubes and centrifuged at 14,490× *g* for 5 min. The results were determined by reading the microhematocrit ruler. Serum cortisol concentrations were analyzed using the chemiluminescence method (Immulite 1000, Siemens Healthineers Diagnostics, Thüringen, Germany). In preparation for the analysis of glucose, LDH, and CK, blood samples were centrifuged at 910× *g* for 10 min, and the supernatant (3 mL) in each tube was collected and split into two microtubes (MaxyClear Axygen^®^, Wujiang, China) and stored at −20 °C until analysis. The plasma glucose concentration was analyzed using the enzymatic colorimetric method (GOD-PAP, Quibasa–Bioclin, Belo Horizonte, MG, Brazil), while serum LDH and CK concentrations were analyzed using a lactate dehydrogenase kit (LDH UV, Quibasa-Bioclin, Belo Horizonte, MG, Brazil) and CK enzymatic kit (Kit CK MB UV, Quibasa-Bioclin, Belo Horizonte, MG, Brazil).

### 2.3. Skin Lesions Scores

Skin lesions were scored on the whole carcass (n = 360 carcasses) in the cooler by a single observer using a subjective 5-point photographic scale (1 = no/mild lesions to 5 = severe deep lesions) [29].

The age of lesions, which helps determine the time of infliction [30], was also determined by the subjective evaluation of color variation, i.e., from red to brown/dark red, corresponding to a fresh and old lesion, respectively (occurred within 10 h and more than 24 h before, respectively), as described by Rocha et al. [31].

### 2.4. Meat Quality Evaluation

At 24 h post-mortem, the loins were transported on ice to the laboratory of the UNIOESTE for meat quality evaluation. Because of the limited storage capacity in the laboratory, the number of loins selected for the meat quality evaluation was reduced to 240 (10 pigs/replicate/treatment). Meat quality was assessed at 24 h post-mortem in the *longissimus lumborum* (LL) muscle (from the second to the last lumbar vertebra) by recording pH (pHu) using a pH meter with a spear-tip pH electrode fitted with an integrated temperature probe (Akso, model AK103, São Leopoldo, RS, Brazil). Color was assessed subjectively using Japanese color standards (from 1 = very pale to 6 = very dark) [32] and objectively using a Minolta CR-400 colorimeter (Konica Minolta Sensing Inc., Osaka, Japan) after exposing the muscle surface to a 30 min blooming period. This latter color measurement was taken using a 10° observer angle, D65 illuminant, SCI (specular component included) mode, and an illumination measurement area of 8 mm in diameter, according to the reflectance coordinates (L*, a*, b*) [33].

Drip loss was evaluated using the gravimetric method, as described by Bridi and Silva [34]. Briefly, one chop (2.5 cm thick, 300 g) was removed from the LL muscle, weighed (scale model LBI WT3000-I-R-ABS, Libratek, Chapecó, SC, Brazil), suspended in a net, placed into a plastic container with no contact with the container sides, and stored for 48 h at 4 °C. At the end of the 48 h storage period, the surface moisture of the muscle chop surface was carefully dabbed, and the chop was reweighed. The drip loss percentage was calculated by dividing the difference between the initial and final chop weights by the initial chop weight.

A further LL muscle chop (2.5 cm thick) was removed from the caudal part (near the last lumbar vertebra) of the LL muscle section and frozen (−20 °C), pending the analysis of thawing and cooking losses and shear force. Frozen samples were weighed (scale model LBI WT3000-I-R-ABS, Libratek, Chapecó, SC, Brazil) before being placed in polyethylene bags and stored in a refrigerator (B.O.D model 347 CD, Fanem, São Paulo, SP, Brazil) at 4 °C for thawing. After 24 h, the samples were removed from the refrigerator (model EL 101/3, Eletrolab, São Paulo, SP, Brazil), dried, and weighed again to determine water loss. They were then left at room temperature for 30 min before being analyzed for water loss by cooking.

Muscle chops were roasted in an electric oven (model Plus, Fischer, Brusque, SC, Brazil) until a final cooking temperature of 71 °C was reached in the core of the sample, as determined with a skewer thermometer (model testo 104, Gewerbestr, Kirchzarten, Germany) inserted in the geometric center of each sample. Samples were then cooled at room temperature and reweighed after refrigerated storage (model EL 101/3, Eletrolab, São Paulo, SP, Brazil) at 4 °C for 24 h to determine the cooking water loss. Later on, for each roasted meat chop, six rectangular cores (1 cm^2^), parallel to the longitudinal orientation of the muscle fibers, were extracted and examined for meat tenderness by the analysis of shear force.

The shear force analysis was carried out using a Warner–Bratzler device attached to a TAXTplus Texture Analyzer (Stable Micro Systems, Godalming, UK). During the analysis, muscle cores were sheared across the fiber axis with a 30 kg cell at a speed of 2 mm/s, and the six readings were averaged.

### 2.5. Statistical Analysis

Data were analyzed according to a randomized complete block design using PROC MIXED and GENMOD from SAS (version 9.3; SAS Inst. Inc., Cary, NC, USA). The statistical model included fixed effects of stocking density, lairage time, their interactions (stocking density × lairage time), and random effects of shipment, week, slaughter time (or slaughter order), and residual error. Ambient temperature was used as a covariate in the model (*p* ≤ 0.05), so that the adjusted means were compared at the average ambient temperature. The lairage pen representing each treatment was considered the experimental unit (n = 24). When a significant interaction was found, a partitioned analysis was carried out using the SLICE statement.

Statistical significance was set at *p* ≤ 0.05 for all tests. Probabilities of 0.05 < *p* ≤ 0.10 were considered tendencies.

## 3. Results

### 3.1. Blood Stress Parameters

Except for blood hematocrit percentage, the interaction stocking density × lairage time had no effect on any blood parameters (*p* > 0.10; Table 2).

When compared to pigs that rested at a lower stocking density (0.66 m^2^/pig), pigs that were kept in lairage at a higher stocking density (0.42 m^2^/pig) tended to have a lower exsanguination blood LDH concentration (2727 vs. 2519 U/L, SEM = 363; *p* = 0.06) and a higher blood cortisol concentration (4.73 vs. 4.00 µg/dL, SEM = 0.70; *p* = 0.08).

Lairage time had no impact on any stress parameters evaluated in the exsanguination blood in this study (*p* > 0.10). However, its interaction with stocking density affected the blood hematocrit percentage, with blood hematocrit percentage being greater (*p* < 0.01) in pigs kept at a lower stocking density (0.66 m^2^/pig) and for a longer time (6 h), compared with the other lairage conditions applied in this study (Table 2).

Neither stocking density nor lairage time influenced the blood concentrations of glucose and CK at slaughter (*p* > 0.10).

### 3.2. Skin Lesion Scores and Meat Quality

Greater skin lesion scores were found on the carcasses of pigs kept in lairage at a higher stocking density, compared to those housed at a lower stocking density (2.17 vs. 2.03, SEM = 0.04; *p* < 0.001; Table 3).

Lairage time also had an impact on skin lesion scores, with the carcasses of pigs that were rested for a longer time (6 h) presenting greater scores, compared to those kept in lairage for a shorter time (2.17 vs. 2.03, SEM=0.07; *p* = 0.02; Table 3).

Separate impacts of stocking density and lairage time were found on lesion color, with a greater proportion of red/fresh lesions than dark lesions being recorded on the carcasses of pigs that rested at a higher stocking density (49.9% vs. 22.3%; *p* < 0.001) and for a longer time (49.9% vs. 22.3%; *p* = 0.01).

Except for pHu and drip loss, neither stocking density nor lairage time had an effect on meat quality traits in this study (*p* > 0.10; Table 3). The LL muscle of pigs kept in lairage for a longer time presented a slightly higher pHu, compared with those rested for a shorter time (5.47 vs. 5.45, SEM=0.20; *p* < 0.01). Drip loss was higher in the LL muscle of pigs kept at a lower stocking density in the lairage pen, compared with those rested at a lower stocking density (4.37% vs. 3.73%, SEM=0.52; *p* = 0.04).

No interaction was found between stocking density and lairage time on skin lesion characteristics and meat quality traits (*p* > 0.10).

## 4. Discussion

A large variation in stocking densities (from 0.28 to 0.73 m^2^/pig or from approx. 1 to 3 pigs/m^2^) and lairage times (ranging from <1 to 24 h) has been recorded in a number of slaughterhouse surveys across the world [17,35,36]. However, their control is recommended to limit aggressions within mixed groups of pigs and to ensure rest aimed at the faster physiological recovery of pigs from the stress of handling and transport and at optimal pork quality production [37].

The results of this study could not confirm the recommendation to adjust stocking density to the time spent by pigs in lairage [20], as, except for the blood hematocrit percentage at slaughter, no interaction between these factors was found.

Our results may confirm the previously reported negative impact of reducing floor space in the pen (from 0.66 to 0.44 m^2^/pig) on pigs’ overlaying to find some room to lie down and rest [38] or fighting activity during lairage [17], as shown by the increased skin lesion scores and greater proportion of red/fresh lesions on the carcasses of these pigs. The greater skin lesion score may explain the higher concentration of cortisol in blood collected at slaughter in these pigs, based on the linear variation between the two parameters reported by Gispert et al. [36] and Warriss et al. [39]. This relationship may be explained by the gradual rise in the psychological–biological response to fear, pain, and fatigue, all resulting from aggressive behavior [40], according to the degree of damage [36,39].

The lower skin lesion score on the carcasses of pigs kept at a lower stocking density may result from both the provision of more floor space for rest and the greater freedom to move and have access to the hanging toys they could play with or explore instead of fighting each other. The presence of enrichment tools (i.e., wood shavings and toys) in the lairage pen has the potential to reduce pigs’ fighting and agonistic interactions and increase exploring or playing [41,42]. A greater proportion of standing and playing pigs was, in fact, observed during the last hour of lairage within this group of pigs in a companion study [38]. These effects, though, appear to depend on the type of manipulable/chewing objects, as recently reported by Sobral et al. [43], who failed to find an impact of hanging a metal chain in the lairage pen on pig behavior and skin lesion scores. However, these enriched housing conditions may also have prevented these pigs from resting properly, as shown by the greater concentration of LDH, an indicator of physical fatigue [44], in their blood collected at slaughter. The greater concentration of LDH in exsanguination blood may have contributed to the slightly higher drip loss in the LL muscle of these pigs, according to the significant, although weak, correlation (*r* = 0.18 to 0.22; *p* < 0.05) between blood lactate and pork meat exudation [22,45].

The hematocrit percentage recorded in exsanguination blood in this study fits within the normal hydration range (32 to 50%) for pigs [46]. However, it increased, as a sign of lower hydration condition at slaughter, in pigs that were kept at a lower stocking density over a longer time before slaughter. This result is hard to explain based on the observations of drinking behavior, which was neither influenced by the stocking density or lairage time nor their interaction [38]. This result confirms the lack of consistent relationship between blood hematocrit percentage and drinking behavior in lairage reported in previous studies [26,47,48].

The increased skin lesion scores recorded on the carcasses of pigs kept in lairage for a longer time (6 vs. 2 h) confirmed the results from a number of previous studies [43,49,50] and may be explained by the combined effects of longer feed deprivation (fasting) and fighting within mixed groups of pigs [12,17,21]. However, contrary to previous studies [39,51,52], lairage time had no effect on the blood profiles of pigs at slaughter.

Lairage significantly influences pork meat quality traits variation [37], due to the concurrent effect of post-transport recovery rate, feed restriction, and fatigue condition on the muscle residual glycogen content at slaughter [22,53]. In a preslaughter study on unmixed pigs, Nanni Costa et al. [54] reported that lairage time (2 h vs. overnight) contributed the most to the variation in meat quality when compared with other preslaughter factors, such as loading by ramp or hydraulic lift and transport with a small or large space allowance (<0.40 vs. >0.60 m^2^/pig). However, the effects of the lairage conditions applied in this study on meat quality were negligible, and overall meat quality was good, probably due to the mild stress conditions experienced by pigs during lairage and/or because the effects of preslaughter physical or psychological stress on meat quality are muscle-dependent, being less evident in postural muscles, such as the LL muscle [22,55,56]. Overall, pHu values were lower than normal, likely due to the effect of the electrolyte-supplemented drinking water [57]. However, they slightly increased in the LL muscle of pigs kept in lairage for a longer time, which can be explained by the combined effects of fasting and fighting (based on the linear relationship between skin lesion scores and muscle pHu [36,39]) on muscle glycogen depletion before slaughter [7,22].

## 5. Conclusions

The recommendation to adjust stocking density to lairage time aimed at reducing aggressions between unfamiliar pigs in the pen and carcass damages could not be validated by the results obtained under the preslaughter conditions of this study. Overall, stocking density, either high or low, had a greater impact on pigs’ stress conditions at slaughter than lairage time. Based on the results of this study, providing pigs with a larger floor space and shorter lairage time reduced skin damage scores. However, this beneficial effect was counteracted by signs of fatigue and dehydration, with the latter being aggravated by a longer lairage time. These results highlight the conflicting effects of keeping pigs in pens enriched with manipulable/chewable materials during lairage. On the one hand, the presence of toys distracted pigs from fighting and other social interactions, contributing to reducing skin lesion scores, and on the other hand, it may stimulate physical activities, such as playing and exploring, to the detriment of resting and drinking.

In conclusion, as resting is the main purpose of lairage, during this phase, mixed groups of pigs should be housed at a lower stocking density in non-enriched pens for 2 h in order to limit the incidence of damaged carcasses and ensure their fitness at slaughter.

However, further research is needed to confirm these conclusions, possibly including longer rest times and space allowances for groups of pigs of the same size under traditionally barren lairage conditions. The assessment of agonistic behaviors in the pen should also be included in further studies in order to better explain the effects of these lairage conditions on lesion scores and colors evaluated during the post-mortem carcass evaluation.

## Figures and Tables

**Table 1:** Distribution of shipments (4 trucks, 1 per combination of lairage treatments) by farm (F) and week.

Farm	Herd Size	Pigs Shipped (n)	Week (n Loads/Shipments)						Total
	(n)		1	2	3	4	5	6	
F1	1600	320	4	0	0	0	0	0	4
F2	3000	320	0	4	0	0	0	0	4
F3	2300	320	0	0	4	0	0	0	4
F4	1500	320	0	0	0	4	0	0	4
F5	4500	640	0	0	0	0	4	4	8

**Table 2:** Effects of stocking density (SD; 0.42 m^2^ and 0.66 m^2^), lairage time (LT; 2 h and 6 h), and their interaction on average blood glucose, creatine kinase (CK), lactate dehydrogenase (LDH), cortisol, and hematocrit concentrations at slaughter.

Parameter	Treatments				SEM	*p*-Value		
	0.42 m^2^		0.66 m^2^					
	2 h	6 h	2 h	6 h		SD	LT	SD × LT
Glucose, mg/dL	165.0	133.6	179.8	133.2	7.65	0.69	0.24	0.43
CK, IU/L	15,157	17,967	16,140	14,340	654.19	0.73	0.79	0.34
LDH, U/L	2728	2726	2785	2254	94.91	0.06	0.20	0.12
Cortisol, µg/dL	4.73	5.16	4.03	3.98	0.16	0.08	0.77	0.52
Hematocrit, %	47.40	47.20	47.40	48.80	0.76	0.76	0.001	<0.01

**Table 3:** Effects of stocking density (SD; 0.42 m^2^ and 0.66 m^2^), lairage time (LT; 2 h and 6 h), and their interaction on skin lesion scores and meat quality traits, as assessed in the *longissimus lumborum* muscle.

Parameter	Treatments				SEM	*p*-Value		
	0.42 m^2^		0.66 m^2^					
	2 h	6 h	2 h	6 h		SD	LT	SD × LT
Skin lesion scores ^1^	2.10	2.24	1.96	2.11	0.02	<0.001	0.02	0.76
pHu	5.48	5.41	5.41	5.54	0.04	0.90	0.05	0.71
JCS ^2^	2.66	2.66	2.66	2.66	0.10	0.41	0.49	0.78
L*	43.7	43.0	44.0	43.1	0.26	0.75	0.81	1.00
a*	3.9	3.6	3.8	3.8	0.07	0.10	0.80	0.75
b*	2.3	2.1	2.2	2.3	0.10	0.37	0.92	0.72
Drip loss, %	3.93	3.52	4.78	3.96	0.18	0.04	0.94	0.42
Thawing loss, %	5.63	5.88	5.69	5.99	0.30	0.59	0.21	0.70
Cooking loss, %	23.77	24.38	23.60	25.38	0.42	0.79	0.94	0.77
WBSF ^3^, kg	3.70	3.68	3.51	3.70	0.07	0.22	0.44	0.43

^1^ Based on the photographic chart (from 1 = none/mild to 5 = severe lesions) [24]; ^2^ based on Japanese color standards (from 1 = pale to 6 = dark) [27]; ^3^ WBSF: Warner–Bratzler shear force.

## Data Availability

Data will be made available upon request.
